# Efficacy, safety and pharmacokinetics of simeprevir and TMC647055/ritonavir with or without ribavirin and JNJ-56914845 in HCV genotype 1 infection

**DOI:** 10.1186/s12876-017-0580-2

**Published:** 2017-02-10

**Authors:** Stefan Bourgeois, Hans Van Vlierberghe, Christophe Moreno, Hans Orlent, Frederik Nevens, Keikawus Arastéh, Yves Horsmans, Jörn M. Schattenberg, Peter Buggisch, Sven Francque, Leen Vijgen, Thomas N. Kakuda, Eva Hoeben, Donghan Luo, An Vandebosch, Bert Jacquemyn, Pieter Van Remoortere, René Verloes

**Affiliations:** 1 Department of Gastroenterology & Hepatology, ZNA Antwerp, Antwerpen, Belgium; 2 Department of Hepatology and Gastroenterology, Universitair Ziekenhuis Gent, Ghent, Belgium; 3 Liver Unit, CUB Hôpital Erasme, Université Libre de Bruxelles, Brussels, Belgium; 4 Department of Gastroenterology and Hepatology, AZ Sint-Jan AV Hospital, Bruges, Belgium; 5 Department of Liver and Biliopancreatic Disorders, University Hospitals KU Leuven, Leuven, Belgium; 6 EPIMED/Vivantes-Auguste-Viktoria-Klinikum, Berlin, Germany; 7 Hepato-gastroenterology, Cliniques Universitaires Saint-Luc, Université Catholique de Louvain, Brussels, Belgium; 8 Department of Medicine, University Medical Center Mainz, Mainz, Germany; 9 IFI, Liver Center Hamburg, Asklepiosklinik St. Georg, Hamburg, Germany; 10 Department of Gastroenterology and Hepatology, Universitair Ziekenhuis Antwerpen, Antwerp, Belgium; 11 Janssen Pharmaceutica NV, Beerse, Belgium; 12 Alios BioPharma Inc, South San Francisco, CA USA; 13 Janssen Research & Development BVBA, Beerse, Belgium; 14 Janssen Pharmaceuticals LLC, Titusville, NJ USA

**Keywords:** Simeprevir, TMC647055/ritonavir, JNJ-56914845, Ribavirin, Direct-acting antiviral agents, Hepatitis C virus, genotype 1, Efficacy, Safety

## Abstract

**Background:**

A Phase 2a, open-label study (NCT01724086) was conducted to assess the efficacy and safety of a once-daily, 2-direct-acting-antiviral-agent (2-DAA) combination of simeprevir + TMC647055/ritonavir ± ribavirin and of the 3-DAA combination of simeprevir + TMC647055/ritonavir + JNJ-56914845 in chronic hepatitis C virus genotype (GT)1-infected treatment-naïve and prior-relapse patients.

**Methods:**

The study comprised four 12-week treatment panels: Panel 1 (*n* = 10; GT1a) and Panel 2-Arm 1 (*n* = 12; GT1b): simeprevir 75 mg once daily + TMC647055 450 mg once daily/ritonavir 30 mg once daily + ribavirin 1000–1200 mg/day; Panel 2-Arm 2 (*n* = 9; GT1b): simeprevir 75 mg + TMC647055 450 mg/ritonavir 30 mg without ribavirin; Panel 3: simeprevir 75 mg + TMC647055 600 mg/ritonavir 50 mg with (Arm 1: GT1a; *n* = 7) or without (Arm 2: GT1b; *n* = 8) ribavirin; Panel 4: simeprevir 75 mg + TMC647055 450 mg/ritonavir 30 mg + JNJ-56914845 30 mg once daily (Arm 1: *n* = 22; GT1a/GT1b) or 60 mg once daily (Arm 2: *n* = 22; GT1a/GT1b). Primary endpoint was sustained virologic response 12 weeks after end of treatment (12 weeks of combination treatment; SVR12).

**Results:**

In Panel 1 and Panel 2-Arm 1, 5/10 and 6/12 (50%) GT1a/GT1b + ribavirin patients achieved SVR12, versus 3/9 (33%) GT1b without ribavirin patients in Panel 2-Arm 2. In Panel 3-Arm 1 and Panel 3-Arm 2, 6/7 (86%) GT1a + ribavirin and 4/8 (50%) GT1b without ribavirin patients, respectively, achieved SVR12. In Panel 4, 10/14 (71%) and 14/15 (93%) GT1a patients in Arms 1 and 2 achieved SVR12 compared with 8/8 and 7/7 (100%) GT1b patients in each arm, respectively. No deaths, serious adverse events (AEs), Grade 4 AEs or AEs leading to treatment discontinuation occurred.

**Conclusions:**

The 2- and 3-DAA combinations were well tolerated. High SVR rates of 93% and 100% in GT1a- and GT1b-infected patients, respectively, were achieved in this study by combining simeprevir with JNJ-56914845 60 mg and TMC647055/ritonavir.

**Trial registration:**

NCT01724086 (date of registration: September 26, 2012)

**Electronic supplementary material:**

The online version of this article (doi:10.1186/s12876-017-0580-2) contains supplementary material, which is available to authorized users.

## Background

Interferon (IFN)-free regimens comprising direct-acting antivirals (DAA) with different mechanisms of action can result in high sustained virologic response (SVR) rates in patients chronically infected with hepatitis C virus (HCV). Combination regimens comprising an HCV NS3/4A protease inhibitor (PI), a non-nucleoside inhibitor (NNI) of the HCV NS5B polymerase and/or an HCV NS5A replication complex inhibitor with/without ribavirin have been shown to be successful in the treatment of chronic HCV infection [1]. For example, the combination of the PI paritaprevir, the NNI dasabuvir and the NS5A inhibitor ombitasvir, with ritonavir included as a pharmacologic booster for paritaprevir, is approved for the treatment of chronic HCV genotype (GT) 1 infection. In the PEARL-III (HCV GT1b) and PEARL-IV (HCV GT1a) Phase 3 studies, treatment of previously untreated patients with paritaprevir/ritonavir, dasabuvir and ombitasvir for 12 weeks resulted in SVR 12 weeks after end of treatment (SVR12) rates of 99.5% and 97.0% in the presence of ribavirin, and 99.0% and 90.2% in the absence of ribavirin, respectively [2].

Simeprevir is a once-daily, HCV NS3/4A PI approved as part of an IFN-free combination with sofosbuvir for HCV GT1 infection. In addition, simeprevir with sofosbuvir is approved for HCV GT4 infection and HCV/human immunodeficiency virus (HIV) co-infection in the European Union (EU). Simeprevir is also approved in combination with pegylated IFN (pegIFN)/ribavirin for chronic HCV GT1 and GT4 infection in the United States and EU [3]. Simeprevir is a cytochrome P450 (CYP) 3A substrate and mild inhibitor (intestinal only). Simeprevir also inhibits organic anion transporting polypeptide (OATP) 1B1/3 and P-glycoprotein [4].

TMC647055 is an NNI of the HCV NS5B polymerase, binding in the NNI-1 pocket on the polymerase. TMC647055 has in vitro antiviral activity against HCV GT1, 3, 4, 5 and 6 [5]. In a Phase 1 study (NCT01202825), TMC647055 1000 mg twice daily in combination with simeprevir 150 mg once daily for 10 days demonstrated good antiviral activity and was well tolerated in HCV GT1a- and GT1b-infected patients [6]. However, the systemic exposure to both compounds decreased during treatment to levels potentially lower than required for complete viral suppression. Co-administering TMC647055 with the potent CYP3A4 inhibitor ritonavir was expected to increase exposure by overcoming CYP3A4 induction [5].

JNJ-56914845 (previously known as GSK-2336805) is a potent HCV NS5A replication complex inhibitor, with in vitro antiviral activity against HCV GT1, 4, 5 and 6a/6b [7, 8]. It is a CYP3A and P-glycoprotein substrate and, in vitro, it inhibits P-glycoprotein, OATP1B1/3 and breast cancer resistance protein (data on file).

This Phase 2a, open-label study (NCT01724086) assessed the efficacy, pharmacokinetics, safety and tolerability of the 2-DAA combination of simeprevir with TMC647055 and a low dose of ritonavir with or without ribavirin for 12 weeks in chronic HCV GT1-infected treatment-naïve and prior-relapse patients. The study also included the addition of a third DAA, JNJ-56914845, to the simeprevir and TMC647055 combination to assess the potential of further increasing the efficacy of the regimen by combining three DAAs with different mechanisms of action. Results from the final analysis, when all patients in all panels had completed the study, are presented here.

## Methods

### Patients and study design

This was a Phase 2a, open-label study, conducted between 12 September 2012 and 16 December 2014 at 12 sites in Belgium and Germany. The study was approved by the Institutional Review Board or Independent Ethics Committee at each participating centre, and met the ethical principles of the Declaration of Helsinki and Good Clinical Practice guidelines. All patients provided written, informed consent.

Adults (18–70 years of age) with chronic HCV GT1a/GT1b infection and screening plasma HCV RNA >10,000 IU/mL who were treatment-naïve or had relapsed following previous treatment with pegIFN/ribavirin were eligible for inclusion. Patients had to have a documented liver biopsy within 3 years of the screening visit, or have an elastography prior to first dosing.

Exclusion criteria included liver cirrhosis, hepatic decompensation, liver disease of non-HCV aetiology, infection/co-infection with non-GT1a/GT1b HCV, hepatitis A or B, or HIV-1/-2, and significant laboratory abnormalities, including total bilirubin ≥1.5 x upper limit of normal and platelet count <90,000/mm^3^. Patients who had received prior HCV-specific DAA treatment or a liver transplant were also excluded.

**Fig. 1:**
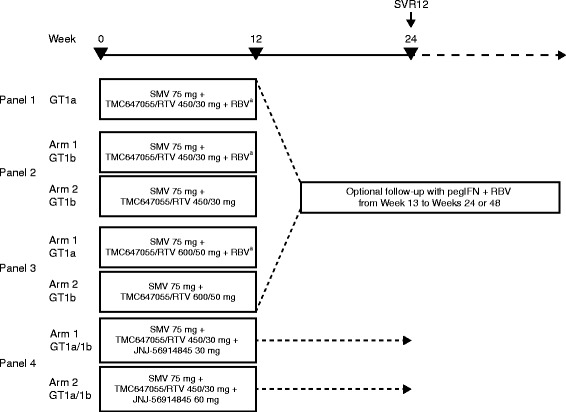
Study design. ^a^Ribavirin given twice daily at a dose of 1000–1200 mg. Follow-up therapy with pegIFN/RBV was based on on-treatment response and was initiated only if: Week 4 HCV RNA ≥25 IU/mL (Panels 1–3: 36 weeks of follow-up therapy); Week 4 HCV RNA <25 IU/mL detectable or HCV RNA confirmed detectable between Week 4 and Week 11 (Panels 1–2: 12 weeks of follow-up therapy). *GT* genotype, *HCV* hepatitis C virus, *pegIFN* pegylated interferon α-2a, *RBV* ribavirin, *RTV* ritonavir, *SMV* simeprevir, *SVR12* sustained virologic response 12 weeks after end of treatment

The study comprised four 12-week treatment panels (Fig. 1). Panels 1 and 2, which ran in parallel, comprised the first part of the study and were sequentially followed by Panels 3 and 4. Panels 1–3 evaluated simeprevir in combination with TMC647055/ritonavir (at two different doses of TMC647055) with ribavirin in GT1a-infected patients and with or without ribavirin in GT1b-infected patients. Panel 4 evaluated the triple combination of simeprevir with TMC647055/ritonavir plus the NS5A inhibitor JNJ-56914845 at a low and high dose in GT1a/b-infected patients.

More specifically, patients in Panels 1 and 2 received simeprevir 75 mg once daily, TMC647055 450 mg once daily, ritonavir 30 mg once daily and ribavirin 1000–1200 mg (patients in Panel 2-Arm 2 did not receive ribavirin). Patients in Panel 3 received simeprevir 75 mg once daily, TMC647055 at a higher dose of 600 mg once daily, ritonavir 50 mg once daily and ribavirin 1000–1200 mg (patients in Panel 3-Arm 2 did not receive ribavirin). Patients in Panel 4 received simeprevir 75 mg once daily, TMC647055 450 mg once daily, ritonavir 30 mg once daily and JNJ-56914845 30 (Arm 1) or 60 mg (Arm 2) once daily. Simeprevir was dosed at 75 mg once daily (rather than the 150 mg dose approved) to account for CYP3A inhibition by ritonavir, which is not a part of the approved regimen for simeprevir. Further details on the dosing rationale are provided in Additional file 1. For ethical reasons, patients in Panels 1–3 received follow-up treatment with pegIFN/ribavirin for an additional 12 or 36 weeks if their Week-4 HCV RNA levels did not comply with predefined criteria (Fig. 1). For patients in Panel 4 receiving the 3-DAA regimen, no follow-up treatment was planned.

Central randomisation was implemented in Panel 2, with patients randomly assigned 1:1 to Arm 1 or Arm 2 based on a computer-generated schedule prepared before the study, balanced by using randomly permuted blocks, and stratified by *IL28B* genotype (CC and non-CC). Patients in Panel 3 were not randomised as their treatment was dependent on their HCV geno/subtype (1a or 1b) (Fig. 1). In Panel 4, patients were randomised in a 1:1 ratio between Arm 1 or Arm 2, stratified by HCV geno/subtype.

To avoid unnecessary drug exposure, the following virologic stopping rules were adopted: all study drugs were discontinued for patients with viral breakthrough (confirmed on-treatment increase of >1 log_10_ IU/mL in HCV RNA from the lowest level reached, or confirmed HCV RNA >100 IU/mL in patients whose HCV RNA had previously been <25 IU/mL), or with inadequate virologic response (confirmed HCV RNA >100 IU/mL at Week 4 or afterwards until Week 11).

Details on protocol deviations are provided in Additional file 1.

### Outcomes

The primary efficacy endpoint was SVR12. Patients achieved SVR12 if they received no pegIFN/ribavirin follow-up treatment after 12 weeks of combination treatment (Panels 1–3 only) and achieved HCV RNA <25 IU/mL undetectable/detectable 12 weeks after actual end of treatment (Panels 1–4). Patients in Panels 1–3 who received follow-up therapy and had HCV RNA <25 IU/mL at 12 weeks after end of treatment were also classed as having achieved SVR12; however, they were considered as failures with regards to the primary endpoint of the study, which focused on the 12-week DAA therapy. The primary safety endpoints included the proportion of patients with adverse events (AEs), serious AEs (SAEs) or abnormal changes in safety-related laboratory values. Secondary endpoints included: the proportion of patients with SVR 24 weeks after end of treatment (SVR24); Week-4 pharmacokinetics of the study drugs; on-treatment virologic failure, including patients with viral breakthrough (defined in the previous section); viral relapse (defined as HCV RNA <25 IU/mL undetectable at the actual end of treatment and confirmed HCV RNA ≥25 IU/mL during post-treatment follow-up); and the presence of HCV NS3/4A, NS5A and/or NS5B variants at baseline and at time of failure in patients not achieving SVR.

### Assessments

Blood samples for HCV RNA level determination were collected at screening and at predefined time points throughout the treatment phase and follow-up period. HCV RNA was measured using the COBAS® TaqMan® HCV Test version 2.0 (Roche Molecular Diagnostics, Pleasanton, CA, USA) for use with the High Pure System assay (lower limit of quantification: 25 IU/mL; limit of detection: 10–15 IU/mL).

HCV geno/subtypes were determined pre-treatment based on sequencing of a part of the NS5B gene (at baseline) when available or by the VERSANT® HCV Genotype 2.0 assay (LiPA) or Trugene HCV Genotyping assay (at screening) (Siemens Healthcare Diagnostics, Erlangen, Germany).

Standard population-based sequencing of the HCV NS3/4A and NS5B regions in Panels 1–3 and of the NS3/4A, NS5A and NS5B regions in Panel 4 was performed at baseline for all patients and post-baseline for patients not achieving SVR12 based on the HCV RNA changes observed in each individual patient and the limits of the sequencing assay.

Plasma pharmacokinetic samples for simeprevir, TMC647055, ritonavir and JNJ-56914845 were collected over 24 h (pre-dose, 1, 2, 3, 4, 5, 6, 8, 10, 12 and 24 h post-dose) at Week 4 from all patients and assayed using validated liquid chromatography-tandem mass spectrometry methods with lower limits of quantification for simeprevir, TMC647055, ritonavir and JNJ-56914845 of 5.0, 5.0, 2.0 and 1.0 ng/mL, respectively (data on file) [9]. Pharmacokinetic parameters including maximum plasma concentration (C_max_) and area under the plasma concentration–time curve over 24 h (AUC_0–24h_) were calculated using non-compartmental analysis (Phoenix WinNonlin® 6.2.1; Certara, Princeton, NJ, USA).

AEs were monitored throughout the treatment phase and follow-up period and up to 24 weeks after actual end of treatment. AEs were coded using the Medical Dictionary for Regulatory Activities (version 16.0 for Panels 1 and 2 and version 17.0 for Panels 3 and 4).

### Statistical analyses

Statistical analyses were performed using SAS® version 9.1 or higher (SAS Institute Inc, Cary, NC, USA).

No formal sample size calculations were performed as this was a proof-of-concept study. For each efficacy endpoint, the proportion of patients was summarised descriptively together with a 95% confidence interval by treatment group and population. The change in log_10_ HCV RNA from baseline at all time points was calculated using descriptive statistics. Pharmacokinetic parameters were calculated using non-compartmental analysis (Phoenix WinNonlin® 6.2.1). All safety and tolerability data were summarised descriptively.

All analyses were conducted using data from the intent-to-treat population, which comprised all patients who received at least one dose of study drug.

## Results

### Patient disposition and baseline characteristics

**Fig. 2:**
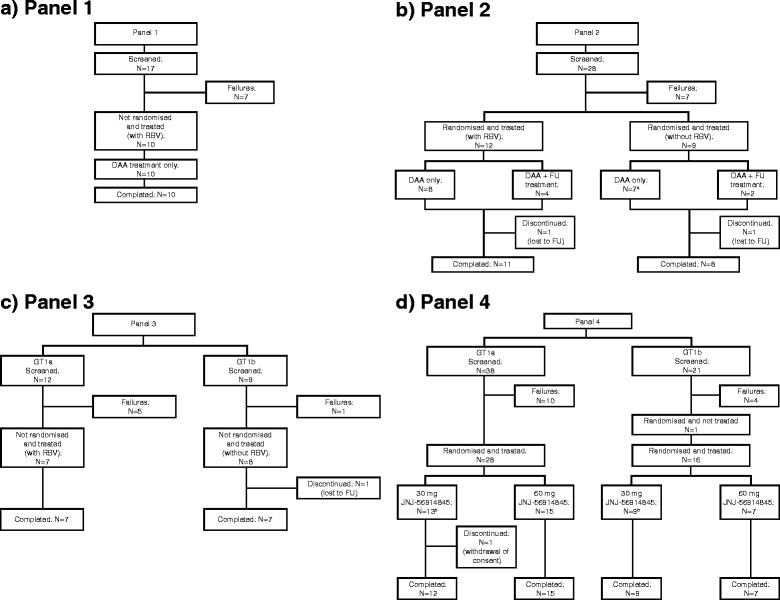
Patient disposition. ^a^In Panel 2, one patient in the GT1b/without RBV group had <25 IU/mL detectable HCV RNA at Week 4 and, therefore, met the criterion for a 12-week follow-up treatment with pegIFN/RBV. This patient refused to receive follow-up treatment. ^b^In Panel 4, the results of the Trugene or LiPA test performed at screening were used to randomise the patients to the 30-mg or 60-mg dose of JNJ-56914845. For analyses purposes, NS5B sequencing was used to determine the HCV geno/subtype. Based on this method, the geno/subtype for two patients in the 30-mg group was found to be non-1a, non-1b (1c, *n* = 1; 1l, *n* = 1) based on geno/subtyping using NS5B sequencing. These two patients were analysed together with the GT1a-infected patients in the category ‘GT1a/other’. *DAA* direct-acting antiviral agent, *FU* follow-up, *GT* genotype, *HCV* hepatitis C virus, *pegIFN* pegylated interferon α-2a, *RBV* ribavirin

In total, 125 patients were screened, and 90 (*n* = 10, 21, 15 and 44 in Panels 1, 2, 3 and 4, respectively) were treated (intent-to-treat population) (Fig. 2). Four (3%) patients discontinued the study prematurely (lost to follow-up, *n* = 3 [Panels 2 and 3]; withdrawal of consent, *n* = 1 [Panel 4]).

In Panel 1, 5/10 (50%) patients completed their treatment regimen. In Panel 2, the majority of patients completed treatment (GT1b/with ribavirin group: 11/12 [92%]; GT1b/without ribavirin group, 8/9 [89%]). Most patients also completed treatment in Panel 3 (GT1a/with ribavirin, 7/7 [100%]; GT1b/without ribavirin, 7/8 [88%]) and Panel 4 (30-mg group, 22/22 [100%]; 60-mg group, 21/22 [96%]). All treatment discontinuations were due to viral breakthrough, as detailed in the next section.

Baseline demographics and disease characteristics are shown in Table 1.

**Table 1:** Baseline patient demographics and disease characteristics (intent-to-treat population)

	Simeprevir 75 mg + TMC647055/ritonavir 450/30 mg			Simeprevir 75 mg + TMC647055/ritonavir 600/50 mg		Simeprevir 75 mg + TMC647055/ ritonavir 450/30 mg + JNJ-56914845 30 mg	Simeprevir 75 mg + TMC647055/ritonavir 450/30 mg + JNJ-56914845 60 mg
	Panel 1	Panel 2		Panel 3		Panel 4	
	GT1a/with ribavirin (*n* = 10)	GT1b/with ribavirin (*n* = 12)	GT1b/without ribavirin (*n* = 9)	GT1a/with ribavirin (*n* = 7)	GT1b/without ribavirin (*n* = 8)	GT1a/b/other (*n* = 22)^a^	GT1a/b/other (*n* = 22)^b^
Caucasian, n (%)	10 (100)	12 (100)	8 (89)	7 (100)	6 (75)	20 (91)	21 (96)
Male, n (%)	8 (80)	4 (33)	7 (78)	7 (100)	3 (38)	16 (73)	17 (77)
Treatment-naïve, n (%)	10 (100)	8 (67)	8 (89)	7 (100)	4 (50)	20 (91)	19 (86)
Prior relapser, n (%)	0 (0)	4 (33)	1 (11)	0 (0)	4 (50)	2 (9)	3 (14)
Age, median, years (range)	46.5 (38–52)	47.5 (29–62)	37.0 (18–64)	44.0 (28–58)	48.5 (43–66)	50.5 (24–70)	48.0 (27–58)
HCV RNA, median, log_10_ IU/mL (range)	6.52 (4.4–7.2)	6.69 (5.8–7.5)	5.98 (5.3–7.1)	6.32 (6.0–7.4)	6.76 (6.1–7.0)	6.78 (5.5–7.4)	6.62 (5.2–7.1)
METAVIR score, n (%)							
F0–F2	5 (50)	6 (50)	6 (67)	4 (29)	3 (38)	7 (32)	5 (23)
F3	1 (10)	1 (8)	1 (11)	1 (14)	0	2 (9)	4 (18)
Missing	4 (40)	5 (42)	2 (22)	2 (29)	5 (63)	13 (59)	13 (59)
*IL28B* genotype, n (%)							
CC	5 (50)	4 (33)	2 (22)	0 (0)	2 (25)	9 (41)	6 (27)
CT	4 (40)	6 (50)	6 (67)	6 (86)	5 (63)	8 (36)	11 (50)
TT	1 (10)	2 (17)	1 (11)	1 (14)	1 (13)	5 (23)	5 (23)

*GT* genotype, *HCV* hepatitis C virus

^a^GT1a/other, *n* = 14; GT1b, *n* = 8. GT1a/other includes one patient with HCV GT1c and one patient with GT1l, as determined by NS5B sequencing

^b^GT1a/other, *n* = 15; GT1b, *n* = 7

### Virologic response

**Fig. 3:**
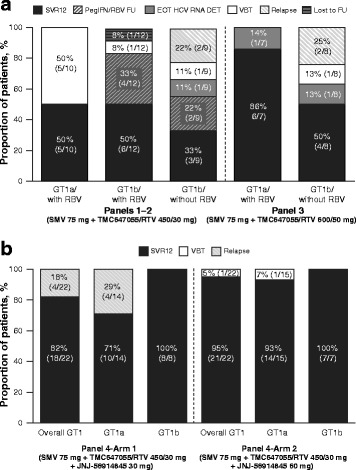
Treatment outcome in (**a**) Panels 1–3, and (**b**) Panel 4. SVR12 data shown represent SVR12 achieved after treatment with only the combination of SMV + TMC647055/RTV ± RBV. In the Panel 2 GT1b/with RBV and GT1b/without RBV arms, four and two patients, respectively, had follow-up therapy with pegIFN/RBV and are, therefore, not included in the SVR12 category that focuses on SVR12 after 12 weeks of DAA treatment. Among them, five patients achieved SVR12 after completing follow-up therapy; one patient refused to receive follow-up treatment and was lost to follow-up. One additional patient in the Panel 2 GT1b/without RBV group had <25 IU/mL detectable HCV RNA at Week 4 and, therefore, met the criterion for the 12-week follow-up treatment with pegIFN/RBV; however, this patient refused to receive follow-up treatment. *DAA* direct-acting antiviral agent*, DET* detectable, *EOT* end of treatment, *FU* follow-up, *GT* genotype, *HCV* hepatitis C virus, *pegIFN* pegylated interferon α-2a, *RBV* ribavirin, *RTV* ritonavir, *SMV* simeprevir, *SVR12* sustained virologic response 12 weeks after end of treatment, *VBT* viral breakthrough

Figure 3 shows the treatment outcome in each of the four panels. In Panel 1, 5/10 (50%) patients achieved SVR12. In Panel 2, SVR12 was achieved in 6/12 (50%) and 3/9 (33%) patients in the GT1b/with ribavirin and GT1b/without ribavirin groups, respectively. In Panel 3, 6/7 (86%) and 4/8 (50%) patients in the GT1a/with ribavirin and GT1b/without ribavirin groups, respectively, achieved SVR12. In Panel 4, SVR12 was achieved in 18/22 (82%) and 21/22 (95%) patients in the JNJ-56914845 30- and 60-mg groups, respectively. In the 30-mg group, SVR12 was achieved in 10/14 (71%) GT1a/other-infected patients and in all (8/8; 100%) GT1b-infected patients. In the 60-mg group, SVR12 was achieved in 14/15 (93%) GT1a/other-infected patients and in all (7/7; 100%) GT1b-infected patients.

All patients who achieved SVR12 also went on to achieve SVR24, with the exception of one patient in Panel 1 and one patient in the GT1b/without ribavirin group in Panel 3 who had HCV RNA ≥25 IU/mL 24 weeks after end of treatment (1,830,000 IU/mL and 2770 IU/mL, respectively).

An overview of treatment outcome, including reasons for not achieving SVR12, is shown in Fig. 3. A total of 5/10 (50%), 1/12 (8%) and 1/9 (11%) patients in Panel 1 and in the GT1b/with ribavirin and GT1b/without ribavirin groups of Panel 2, respectively, experienced viral breakthrough. In Panel 3, 1/8 (13%) patients in the GT1b/without ribavirin group had viral breakthrough compared with 1/22 (5%) patients in the 60-mg group of Panel 4 (Fig. 3). Among the patients with undetectable HCV RNA at end of treatment and with at least one post-treatment follow-up HCV RNA measurement available, 2/6 patients (33%) in each of the Panel 2 and Panel 3 GT1b/without ribavirin groups and 4/14 (18%) GT1a-infected patients in the Panel 4 30-mg group experienced viral relapse. At the time of the final analysis, one patient in Panel 1 and one patient in the Panel 3 GT1b/without ribavirin group achieved SVR12 but had HCV RNA ≥25 IU/mL 24 weeks after end of treatment.

### Virologic resistance testing

At baseline, the NS3 Q80K polymorphism was observed in 2/10 (20%) GT1a-infected patients in Panel 1 and in 2/13 (15%) GT1a-infected patients in the 30-mg group in Panel 4. Neither of the two GT1a-infected patients with baseline Q80K in Panel 1, and one of the two patients in Panel 4 achieved SVR12. In 5/42 (12%) patients with NS5A sequencing data available in Panel 4, baseline NS5A polymorphisms were detected at positions 28, 30, 31 and/or 93. Of those, only L31M (*n* = 1) and Y93C (in combination with M28V; *n* = 1), both detected in GT1a-infected patients, are associated with in vitro resistance to JNJ-56914845 [8]. Details on NS5B baseline polymorphisms are provided in Additional file 1.

Virologic failure (including the two patients who achieved SVR12 but had HCV RNA ≥25 IU/mL 24 weeks after end of treatment) was, in the majority of patients, associated with the emergence of simeprevir and TMC647055 resistance-associated variants (RAVs) in Panels 1–3 and simeprevir and JNJ-56914845 RAVs with or without TMC647055 RAVs in Panel 4 at time of failure. In line with the previously characterised simeprevir resistance profile, most GT1b-infected patients harboured an emerging mutation at NS3 position 168, while emerging mutations in GT1a-infected patients were mostly observed at NS3 position 155 (R155K alone or in combination with a Q80R; R155S) at time of failure. TMC647055 RAVs were only observed at NS5B position 495 (mainly P495L), consistent with the resistance profile of NNI-1 polymerase inhibitors. In all patients with virologic failure in Panel 4, emerging JNJ-56914845 RAVs at NS5A positions 30 and/or 31 were detected at time of failure. Treatment-emergent RAVs in NS3 and NS5B became undetectable in many of the patients after treatment was stopped, while emerging RAVs in NS5A could still be detected by population sequencing at the end of the study in the five patients who failed the 3-DAA treatment.

### Pharmacokinetics

Co-administration of high-dose TMC647055/ritonavir (600/50 mg) increased simeprevir exposure compared with low-dose TMC647055/ritonavir (450/30 mg). Simeprevir C_max_ values were increased by 2.0-fold and AUC_0–24h_ values by 1.9- to 2.3-fold, respectively, following co-administration with TMC647055/ritonavir (600/50 mg vs 450/30 mg) with ribavirin (Panel 3-Arm 1 vs Panel 1) or without ribavirin (Panel 3-Arm 2 vs Panel 2-Arm 2) (Additional file 2: Figure S1a).

A more than dose-proportional increase in TMC647055 exposure occurred following co-administration of TMC647055/ritonavir 600 mg/50 mg once daily (Panel 3-Arm 2) compared with 450 mg/30 mg once daily (Panel 2-Arm 2) with simeprevir (C_max_: 2.6-fold increase; AUC_0–24h_: 2.4-fold increase) (Additional file 2: Figure S1b).

Co-administration of high-dose JNJ-56914845 60 mg once daily with simeprevir 75 mg once daily + TMC647055/ritonavir 450 mg/30 mg once daily increased simeprevir exposure (1.2-fold increase in both C_max_ and AUC_0–24h_ vs without JNJ-56914845) (Additional file 2: Figure S1a), but had minimal effect on TMC647055 and ritonavir exposure (Additional file 2: Figure S1b and c).

A dose-proportional increase in exposure to JNJ-56914845 60 mg versus 30 mg once daily was observed when co-administered with simeprevir 75 mg + TMC647055/ritonavir 450 mg/30 mg once daily (Panel 4-Arm 2 vs Panel 4-Arm 1: C_max_ 821 vs 389 ng/mL, AUC_0–24h_ 7747 vs 3358 ng · h/mL) (Additional file 2: Figure S1d; Additional file 3: Table S1).

No differences were observed in simeprevir, TMC647055 or ritonavir pharmacokinetics based on ribavirin use (Additional file 4: Table S2, Additional file 5: Table S3 and Additional file 6: Table S4).

### Safety

No patients discontinued any of the study drugs due to an AE, and there were no SAEs or deaths.

The incidence of AEs was similar between Panels 1 and 2, and Panel 3 (Table 2). In Panels 1 and 2, all reported AEs were Grade 1 or 2, and there were no clinically relevant differences among patients treated with ribavirin and those who did not receive ribavirin in terms of the incidence or severity of AEs. In Panel 3, all AEs were Grade 1 or 2, with the exception of one patient (GT1b/without ribavirin group) who experienced a Grade 3 AE (hypercholesterolaemia). This AE was not serious and was not considered related to simeprevir, TMC647055 or ritonavir by the investigator; the AE was ongoing at the time of the final analysis. A higher incidence of patients in Panel 3 treated with ribavirin experienced a Grade 1 or 2 AE compared with those who did not receive ribavirin. All AEs in Panel 4 were Grade 1 or 2, with the exception of one patient who experienced a Grade 3 increase in white blood cell count (not related to any study drug). There were no clinically relevant differences in the incidence of AEs between patients treated with JNJ-56914845 at either dose (Table 2).

**Table 2:** Summary of adverse events during the treatment phase (intent-to-treat population)

	Simeprevir 75 mg + TMC647055/ritonavir 450/30 mg			Simeprevir 75 mg + TMC647055/ritonavir 600/50 mg		Simeprevir 75 mg + TMC647055/ritonavir 450/30 mg + JNJ-56914845 30 mg	Simeprevir 75 mg + TMC647055/ritonavir 450/30 mg + JNJ-56914845 60 mg
	Panel 1	Panel 2		Panel 3		Panel 4	
	GT1a/with ribavirin (*n* = 10)	GT1b/with ribavirin (*n* = 12)	GT1b/without ribavirin (*n* = 9)	GT1a/with ribavirin (*n* = 7)	GT1b/without ribavirin (*n* = 8)	GT1a/b/other (*n* = 22)^a^	GT1a/b/other (*n* = 22)^b^
Any AE, n (%)	10 (100)	12 (100)	7 (78)	6 (86)	6 (75)	20 (91)	22 (100)
Worst Grade 1, n (%)	6 (60)	7 (58)	4 (44)	5 (71)	3 (38)	15 (68)	15 (68)
Worst Grade 2, n (%)	4 (40)	5 (42)	3 (33)	1 (14)	2 (25)	5 (23)	6 (27)
Worst Grade 3, n (%)	0 (0)	0 (0)	0 (0)	0 (0)	1 (13)^c^	0 (0)	1 (5)^d^
Worst Grade 4, n (%)	0 (0)	0 (0)	0 (0)	0 (0)	0 (0)	0 (0)	0 (0)
Leading to permanent stop of study drugs, n (%)	0 (0)	0 (0)	0 (0)	0 (0)	0 (0)	0 (0)	0 (0)
Serious AE, n (%)	0 (0)	0 (0)	0 (0)	0 (0)	0 (0)	0 (0)	0 (0)
Death, n (%)	0 (0)	0 (0)	0 (0)	0 (0)	0 (0)	0 (0)	0 (0)

*AE* adverse event, *GT* genotype

^a^GT1a/other, *n* = 14; GT1b, *n* = 8. GT1a/other includes one patient with HCV GT1c and one patient with GT1l, as determined by NS5B sequencing

^b^GT1a/other, *n* = 15; GT1b, *n* = 7

^c^Hypercholesterolaemia, not related to any study drug

^d^Increase in white blood cell count, not related to any study drug

The most frequently (in >20% of patients) reported AEs during the treatment phase were as follows; Panels 1 and 2: headache (14/31 [45%]), fatigue (9/31 [29%]) and influenza-like illness (7/31 [23%]); Panel 3: nausea (7/15 [47%]), headache (7/15 [47%]), diarrhoea (5/15 [33%]), fatigue (4/15 [27%]) and pruritus (4/15 [27%]); Panel 4: headache (15/44 [33%]), diarrhoea (14/44 [32%]) and fatigue (13/44 [30%]).

The majority of graded laboratory abnormalities were Grade 1 or 2 and were not clinically significant. Grade 3 or 4 laboratory abnormalities were observed in <10% of patients in all panels (further details are provided in Additional file 1). Hyperbilirubinaemia was reported as a laboratory-related AE in 1 (3%) patient in Panels 1 and 2 (considered possibly related to TMC647055 and simeprevir) and 1 (2%) patient in the 60-mg group in Panel 4 (considered possibly related to TMC647055, ritonavir and JNJ-56914845, and probably related to simeprevir). Both hyperbilirubinaemia AEs were Grade 1 or 2 in severity.

## Discussion

This was a proof-of-concept study performed to explore the efficacy and safety of the 2-DAA combination of the HCV NS3/4A PI simeprevir and the HCV NS5B NNI TMC647055 administered with ritonavir ± ribavirin, and of the 3-DAA combination of simeprevir, TMC647055 and the HCV NS5A inhibitor JNJ-56914845 administered with ritonavir in HCV GT1-infected patients. The results suggest that the addition of JNJ-56914845 (60 mg) to the 2-DAA regimen of simeprevir and TMC647055 was highly beneficial in improving the efficacy of the combination treatment in patients with HCV GT1 infection.

Overall, the 2-DAA combination of low-dose TMC647055/ritonavir plus simeprevir, with or without ribavirin, did not demonstrate high efficacy in GT1-infected patients (SVR12: 33%–50%). The SVR12 rate in HCV GT1a-infected patients treated with the 2-DAA combination and ribavirin was 86% (6/7) for the high dose of TMC647055/ritonavir, and 50% (5/10) for the low dose of TMC647055/ritonavir, indicating a dose-dependent increase in efficacy of TMC647055/ritonavir. The increased dose was not associated with an increase in AEs and there were no safety concerns regarding the use of ritonavir. Although the 2-DAA combination and the high dose of TMC647055/ritonavir demonstrated a higher SVR12 rate in GT1a-infected patients compared with the low dose of TMC647055/ritonavir, this was not observed in GT1b-infected patients who received the 2-DAA combination without ribavirin (SVR12 for the low- vs high-dose TMC647055/ritonavir: 33% [3/9] vs 50% [4/8], respectively). Although data are limited, these suggest the addition of ribavirin may be required to increase SVR12 in a 2-DAA regimen combining an NS3/4A PI and an NS5B NNI.

In Panel 1, neither of the two GT1a-infected patients with an NS3 Q80K polymorphism at baseline achieved SVR12. Moreover, viral breakthrough was frequently observed with the 2-DAA combination (ranging from 8–50%) compared with a frequency of 5% with the 3-DAA combination (1/22 patient who received the 3-DAA combination with JNJ-56914845 [60 mg]).

The 3-DAA combination resulted in an overall SVR12 rate of 95% with 60 mg JNJ-56914845. All GT1b-infected patients (7/7; 100%) treated with the 3-DAA combination achieved SVR12, and the majority of GT1a-infected patients (14/15; 93%) also achieved SVR12 following treatment with this regimen. The SVR12 rates observed with this combination therapy are consistent with those reported in Phase 3 studies that assessed the combination of the PI paritaprevir with the NNI dasabuvir and the NS5A inhibitor ombitasvir in treatment-naïve or treatment-experienced, non-cirrhotic, HCV GT1-infected patients [2, 10–12].

The simeprevir resistance profile observed in all panels in this study was similar to that observed in the simeprevir Phase 3 studies in the presence of pegIFN/ribavirin [13–15]. A genotypic and phenotypic analysis of baseline HCV isolates from all patients who participated in this study, as well as of isolates obtained at the time of failure and at the end of study from patients with virologic failure, are described in full in a separate paper (Leen Vijgen, Kim Thys, An Vandebosch, Pieter Van Remoortere, René Verloes, Sandra De Meyer. Virology analysis in HCV genotype 1-infected patients treated with the combination of simeprevir and TMC647055/ritonavir, with and without ribavirin, and JNJ-56914845, in preparation).

The interaction between the drugs assessed in this study had previously been evaluated in healthy volunteers [16]. In that study, co-administration of simeprevir increased exposure (AUC_0–24h_) of JNJ-56914845 2.6-fold with no additional effect when TMC647055/ritonavir was added. Co-administration with JNJ-56914845 resulted in small increases in the plasma concentrations of simeprevir and TMC647055. These increases were not considered to be clinically relevant for either DAA [16]. In the current study, administration of simeprevir 75 mg once daily in combination with TMC647055/ritonavir in HCV-infected patients resulted in simeprevir exposures within the range observed in historical data for simeprevir when dosed at 150 mg once daily (data on file). In addition, high exposure to TMC647055 was achieved when administered in combination with ritonavir.

Both the 2-DAA and the 3-DAA regimens were well tolerated. There were no deaths, SAEs, Grade 4 AEs or AEs leading to temporary/permanent treatment discontinuation in any of the four panels. In addition, there were no Grade 3 or 4 laboratory-related AEs that were considered related to any of the study drugs. Differences in AE incidence among patients who received ribavirin and those who did not in Panels 1 and 2 (Table 2) were a result of ribavirin-related adverse drug reactions, as were the respective differences observed in the incidence of laboratory abnormalities in Panels 1–3 (see Additional file 1). A comparable safety profile was observed with TMC647055/ritonavir 450/30 mg and TMC647055/ritonavir 600/50 mg (Panels 1–3) and also between the JNJ-56914845 30-mg and 60-mg doses (Panel 4).

## Conclusion

High SVR rates of 93% and 100% in GT1a- and GT1b-infected patients, respectively, were achieved in this study by combining simeprevir 75 mg with JNJ-56914845 60 mg and TMC647055/ritonavir 450/30 mg. Lower SVR rates were observed without the inclusion of JNJ-56914845. Based on the results of this study, the efficacy and safety of DAA-combination regimens targeting the same viral proteins as the drugs in this study (i.e., the HCV NS5B polymerase, the HCV NS5A protein and the HCV NS4/4A protease) are being investigated further.

## Additional files

Additional file 1: Further information on the methods of the study, including dosing rationale and protocol deviations are included. In addition, further virologic resistance testing and safety results are included. (DOCX 16 kb)
Additional file 2:
**Figure S1.** Mean (SD) plasma concentration versus time profile for: (a) simeprevir; (b) TMC647055; and (c) ritonavir in Panels 1–4; and for (d) JNJ-56914845 in Panel 4. *RBV* ribavirin, *RTV* ritonavir, *SD* standard deviation, *SMV* simeprevir. (DOCX 564 kb)
Additional file 3:
**Table S1.** Week-4 JNJ-56914845 pharmacokinetic parameters after administration in Panel 4. (DOCX 14 kb)
Additional file 4:
**Table S2.** Week-4 simeprevir pharmacokinetic parameters after administration in (a) Panels 1–3 and (b) Panel 4. (DOCX 15 kb)
Additional file 5:
**Table S3.** Week-4 TMC647055 pharmacokinetic parameters after administration in (a) Panels 1–3 and (b) Panel 4. (DOCX 15 kb)
Additional file 6:
**Table S4.** Week-4 ritonavir pharmacokinetic parameters after administration in (a) Panels 1–3 and (b) Panel 4. (DOCX 15 kb)

## Abbreviations

AE: Adverse event
AUC_0–24h_: Area under the plasma concentration–time curve over 24 h
BID: Twice daily
BQL: Below quantification limit
C_max_: Maximum plasma concentration
C_min_: Minimum plasma concentration
CYP: Cytochrome P450
DAA: Direct-acting-antiviral agent
DET: Detectable
EOT: End of treatment
EU: European Union
FU: Follow-up
GT: Genotype
HCV: Hepatitis C virus
HIV: Human immunodeficiency virus
IFN: Interferon
ITT: Intent-to-treat
LDL: Low-density lipoprotein
MCHC: Mean corpuscular haemoglobin concentration
NNI: Non-nucleoside inhibitor
OATP: Organic anion transporting polypeptide
pegIFN: Pegylated interferon α-2a
PI: Protease inhibitor
RAV: Resistance-associated variant
RBV: Ribavirin
RTV: Ritonavir
SAE: Serious adverse event
SD: Standard deviation
SMV: Simeprevir
SVR: Sustained virologic response
SVR12: Sustained virologic response 12 weeks after end of treatment
SVR24: Sustained virologic response 24 weeks after end of treatment
VBT: Viral breakthrough
WBC: White blood cell
