# Supplementary material for: Efficacy, safety and pharmacokinetics of simeprevir and TMC647055/ritonavir with or without ribavirin and JNJ-56914845 in HCV genotype 1 infection
Source: BMC Gastroenterol. 2017 Feb 10;17:26. doi: 10.1186/s12876-017-0580-2 (PMC5303260; doi:10.1186/s12876-017-0580-2)
Supplement: Additional file 1: — Further information on the methods of the study, including dosing rationale and protocol deviations are included. In addition, further virologic resistance testing and safety results are included. (DOCX 16 kb) [file 12876_2017_580_MOESM1_ESM.docx]

# Additional file 1

## Methods

### Dosing rationale

The pharmacokinetics, safety and tolerability of the combination of simeprevir, TMC647055 and ritonavir at doses defined using physiologically based pharmacokinetic modelling were evaluated in the TMC647055HPC1006 study in healthy volunteers (data on file). The volunteers received simeprevir 50 mg once daily, TMC647055 300 mg once daily and ritonavir 20 mg once daily for 14 days during the first combination treatment period and simeprevir 100 mg once daily, TMC647055 600 mg once daily and ritonavir
30 mg once daily for 14 days during the second combination treatment period. Additional doses were tested so that the plasma exposure (area under the plasma concentration–time curve) of the two direct-acting antiviral agents was similar to that achieved with doses previously found to be generally safe and efficacious in hepatitis C virus (HCV)-infected patients treated with simeprevir in combination with pegylated interferonα-2a (pegIFN)/ribavirin (i.e., 150 mg once daily for simeprevir) and as 1000 mg twice daily (BID) for TMC647055 monotherapy. Based on the results of a previous Phase 1 study, the dosing regimen of simeprevir, TMC647055 and ritonavir in this study was determined.

JNJ-56914845 exposure was shown to increase upon co-administration with the simeprevir/TMC647055/ritonavir combination. In healthy volunteers, JNJ-56914845 exposure was increased about 1.5-fold upon co-administration of JNJ-56914845 30 mg and ritonavir 100 mg BID (data on file). A higher increase in exposure, for example up to 3-fold, would still have allowed an acceptable safety margin. Thus, a dose of JNJ-56914845 60 mg once daily (a dose found to be generally well tolerated in HCV-infected patients when administered in combination with pegIFN/ribavirin over a 28-day treatment period [data on file]) was considered to be safe in combination with simeprevir, TMC647055 and ritonavir.

Safety, pharmacokinetic and HCV RNA data from Panels 1 and 2, which ran prior to Panels 3 and 4, indicated that the combination of simeprevir with TMC647055 and ritonavir with and without ribavirin was generally safe and well tolerated, and that exploration of higher doses of TMC647055 and ritonavir might optimise the pharmacokinetic profile and efficacy of the regimen. Based on this, patients in Panel 3 received an increased dose of TMC647055/ritonavir.

### Protocol deviations

In Panel 1, 5/10 (50 %) patients had at least one major protocol deviation which was related to taking ritonavir past the expiry date, or missing planned assessments/visits. In Panel 2, 9/21 (43 %) patients had at least one major protocol deviation: 1 (5 %) patient in the genotype (GT)1b/without ribavirin group did not have evidence of documented chronic GT1a or GT1b HCV infection, and 8 (38 %) patients (GT1b/with ribavirin and GT1b/without ribavirin groups) took ritonavir past the expiry date or missed planned visits. In Panel 3, 3/15 (20 %) patients had at least one major protocol deviation: in the GT1a/with ribavirin group, 1 (7 %) patient did not have a normal 12-lead electrocardiograph at screening, and 1 (7 %) patient received disallowed concomitant medication (trazodone for the adverse event, somnolence); in the GT1b/without ribavirin group, 1 (7 %) patient had missing visits. In Panel 4, 10 (23 %) patients had at least one major protocol deviation: 6 (28 %) and 4 (18 %) patients in the 30- and 60-mg groups, respectively (1 [2 %] patient received the wrong treatment of incorrect dose; 9 [21 %] patients took ritonavir past the expiry date; were not compliant with intake and return of TMC647055, simeprevir and JNJ-56914845; did not attend planned assessments/visits; or were included in the study prior to having the creatinine clearance result available).

# Results

### Virologic resistance testing

NS5B polymorphisms were detected at baseline at the non-nucleoside inhibitor (NNI)-1 binding pocket positions 37, 392, 424, 425, 494 and/or 499 across all panels. None of the polymorphisms observed at baseline, for which *in vitro* site-directed mutagenesis data were available, were found to be associated with reduced *in vitro* susceptibility to TMC647055 (fold change in 50 % effective concentration compared to wild-type replicon <2).

### Safety

In Panels 1 and 2, the most frequently (in >20 % of patients) observed graded laboratory abnormalities were: hyperbilirubinaemia (11/31 [35 %]); fibrinogen high (10/31 [32 %]); cholesterol increased (9/31 [29 %]); low-density lipoprotein (LDL) cholesterol increased (8/31 [26 %]); alanine transaminase increased (7/31 [23 %]); and white blood cell (WBC) decreased (7/31 [23 %]). In Panel 3, the most frequently observed graded laboratory abnormalities were: fibrinogen high (5/15 [33 %]); LDL cholesterol increased (4/15 [27 %]); and WBC increased (4/15 [27 %]). In Panel 4, the most frequently observed graded laboratory abnormalities were cholesterol increased (23/44 [52 %]); LDL cholesterol increased (20/44 [45 %]); fibrinogen high (16/44 [36 %]); and WBC increased (11/44 [25 %]).

In Panels 1 and 2, among patients with and without ribavirin in their regimen, the following differences in laboratory abnormalities were observed: haematocrit below normal was observed in 12 (55 %) versus 0 patients; mean corpuscular haemoglobin concentration (MCHC) below normal and WBC decreased both in 7 (32 %) versus 0 patients; and erythrocytes below normal in 21 (95 %) and 1 (11 %) patients, respectively. Similarly, in Panel 3, among patients with and without ribavirin in their treatment regimen: haematocrit below normal was observed in 5 (71 %) versus 0 of the patients; MCHC below normal in 3 (43 %) versus 1 (13 %) patient; and haemoglobin decreased in 2 (29 %) versus 0 of the patients, respectively.
