# Supplementary material for: Efficacy, safety and pharmacokinetics of simeprevir and TMC647055/ritonavir with or without ribavirin and JNJ-56914845 in HCV genotype 1 infection
Source: BMC Gastroenterol. 2017 Feb 10;17:26. doi: 10.1186/s12876-017-0580-2 (PMC5303260; doi:10.1186/s12876-017-0580-2)
Supplement: Additional file 2: — Figure S1. Mean (SD) plasma concentration versus time profile for: (a) simeprevir; (b) TMC647055; and (c) ritonavir in Panels 1–4; and for (d) JNJ-56914845 in Panel 4. RBV ribavirin, RTV ritonavir, SD standard deviation, SMV simeprevir. (DOCX 564 kb) [file 12876_2017_580_MOESM2_ESM.docx]

**Additional file 2: Figure S1** Mean (SD) plasma concentration versus time profile for: (**a**) simeprevir; (**b**) TMC647055; and (**c**) ritonavir in Panels 1–4; and for (**d**) JNJ-56914845 in Panel 4


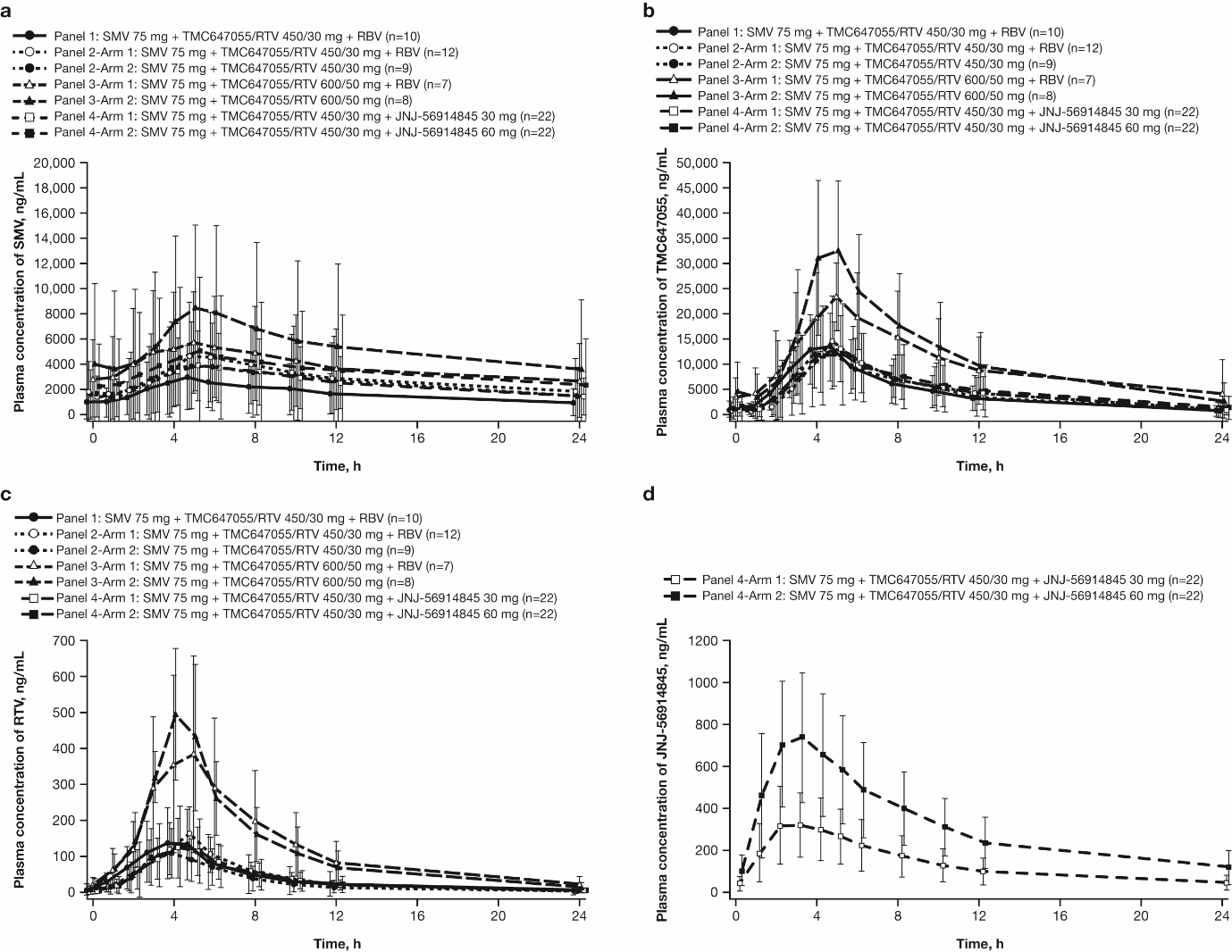


*RBV* ribavirin, *RTV* ritonavir, *SD* standard deviation, *SMV* simeprevir
