# Supplementary material for: Efficacy, safety and pharmacokinetics of simeprevir and TMC647055/ritonavir with or without ribavirin and JNJ-56914845 in HCV genotype 1 infection
Source: BMC Gastroenterol. 2017 Feb 10;17:26. doi: 10.1186/s12876-017-0580-2 (PMC5303260; doi:10.1186/s12876-017-0580-2)
Supplement: Additional file 3: — Table S1. Week-4 JNJ-56914845 pharmacokinetic parameters after administration in Panel 4. (DOCX 14 kb) [file 12876_2017_580_MOESM3_ESM.docx]

**Additional file 3: Table S1** Week-4 JNJ-56914845 pharmacokinetic parameters after administration in Panel 4

|  | Simeprevir 75 mg + TMC647055/ritonavir 450/30 mg + JNJ-56914845 30 mg | Simeprevir 75 mg + TMC647055/ritonavir  450/30 mg + JNJ-56914845 60 mg |
| --- | --- | --- |
|  | Panel 4 | |
| Mean ± SD | GT1a/b/other (*n* = 22)^a^ | GT1a/b/other (*n* = 22) |
| C_min_, ng/mL | 40.3 ± 33.3 | 96.3 ± 7.07 |
| C_max_, ng/mL | 389 ± 171 | 821 ± 298 |
| AUC_0–24h_, ng⋅h/mL | 3358 ± 1769 | 7747 ± 3452 |

*AUC_0–24h_* area under the plasma concentration–time curve over 24 hours, *C_max_* maximum plasma concentration, *C_min_* minimum plasma concentration, *GT* genotype, SD, standard deviation
^a^*n* = 21 for AUC_0–24h_
