# Supplementary material for: Efficacy, safety and pharmacokinetics of simeprevir and TMC647055/ritonavir with or without ribavirin and JNJ-56914845 in HCV genotype 1 infection
Source: BMC Gastroenterol. 2017 Feb 10;17:26. doi: 10.1186/s12876-017-0580-2 (PMC5303260; doi:10.1186/s12876-017-0580-2)
Supplement: Additional file 4: — Table S2. Week-4 simeprevir pharmacokinetic parameters after administration in (a) Panels 1–3 and (b) Panel 4. (DOCX 15 kb) [file 12876_2017_580_MOESM4_ESM.docx]

**Additional file 4: Table S2** Week-4 simeprevir pharmacokinetic parameters after administration in (**a**) Panels 1–3 and (**b**) Panel 4

**a**

|  | Simeprevir 75 mg + TMC647055/ritonavir 450/30 mg | | | Simeprevir 75 mg + TMC647055/ritonavir 600/50 mg | |
| --- | --- | --- | --- | --- | --- |
|  | Panel 1 | Panel 2 | | Panel 3 | |
| Mean ± SD | GT1a/with ribavirin (*n* = 10) | GT1b/with ribavirin (*n* = 12) | GT1b/without ribavirin (*n* = 9)^a^ | GT1a/with ribavirin (*n* = 7) | GT1b/without ribavirin (*n* = 8) |
| C_min_, ng/mL | 891 ± 1094 | 1566 ± 2178 | 1294 ± 1923 | 2545 ± 2922 | 3399 ± 5550 |
| C_max_, ng/mL | 3043 ± 2509 | 5280 ± 4157 | 4583 ± 3691 | 5989 ± 4367 | 8966 ± 6509 |
| AUC_0–24h_, ng⋅h/mL | 40,064 ±  38,489 | 67,612 ±  67590 | 67,437 ±  76,272 | 91,627 ± 82,074 | 127,318 ± 148,663 |

**b**

|  | Simeprevir 75 mg + TMC647055/ritonavir 450/30 mg + JNJ-56914845 30 mg | Simeprevir 75 mg + TMC647055/ritonavir 450/30 mg + JNJ-56914845 60 mg |
| --- | --- | --- |
|  | Panel 4 | |
| Mean ± SD | GT1a/b/other (*n* = 22)^b^ | GT1a/b/other (*n* = 22) |
| C_min_, ng/mL | 1097 ± 1266 | 1962 ± 3170 |
| C_max_, ng/mL | 4240 ± 2799 | 5419 ± 6166 |
| AUC_0–24h_, ng⋅h/mL | 59,307 ± 48,818 | 80,893 ± 103,010 |

*AUC_0–24h_* area under the plasma concentration–time curve over 24 hours, *C_max_* maximum plasma concentration, *C_min_* minimum plasma concentration, *GT* genotype, SD, standard deviation
^a^*n* = 8 for C_max_ and AUC_0–24h_
^b^*n* = 21 for AUC_0–24h_
