# Supplementary material for: Efficacy, safety and pharmacokinetics of simeprevir and TMC647055/ritonavir with or without ribavirin and JNJ-56914845 in HCV genotype 1 infection
Source: BMC Gastroenterol. 2017 Feb 10;17:26. doi: 10.1186/s12876-017-0580-2 (PMC5303260; doi:10.1186/s12876-017-0580-2)
Supplement: Additional file 5: — Table S3. Week-4 TMC647055 pharmacokinetic parameters after administration in (a) Panels 1–3 and (b) Panel 4. (DOCX 15 kb) [file 12876_2017_580_MOESM5_ESM.docx]

**Additional file 5: Table S3** Week-4 TMC647055 pharmacokinetic parameters after administration in (**a**) Panels 1–3 and (**b**) Panel 4

**a**

|  | Simeprevir 75 mg + TMC647055/ritonavir 450/30 mg | | | Simeprevir 75 mg + TMC647055/ritonavir 600/50 mg | |
| --- | --- | --- | --- | --- | --- |
|  | Panel 1 | Panel 2 | | Panel 3 | |
| Mean ± SD | GT1a/with ribavirin (*n* = 10) | GT1b/with ribavirin (*n* = 12) | GT1b/without ribavirin (*n* = 9)^a^ | GT1a/with ribavirin (*n* = 7) | GT1b/without ribavirin (*n* = 8) |
| C_min_, ng/mL | 670 ± 1071 | 695 ± 1275 | 798 ± 1464 | 2710 ± 3825 | 2591 ± 3594 |
| C_max_, ng/mL | 14,626 ± 6619 | 15,029 ± 9034 | 13,936 ± 6228 | 23,714 ± 7255 | 36,288 ± 15,229 |
| AUC_0–24h_, ng⋅h/mL | 103,452 ± 61,231 | 109,672 ± 77,498 | 112,769 ± 84,867 | 237,623 ± 162,469 | 273,964 ± 165,632 |

**b**

|  | Simeprevir 75 mg + TMC647055/ritonavir 450/30 mg + JNJ-56914845 30 mg | Simeprevir 75 mg + TMC647055/ritonavir  450/30 mg + JNJ-56914845 60 mg |
| --- | --- | --- |
|  | Panel 4 | |
| Mean ± SD | GT1a/b/other (*n* = 22)^b^ | GT1a/b/other (*n* = 22) |
| C_min_, ng/mL | 655 ± 826 | 1159 ± 2097 |
| C_max_, ng/mL | 14,189 ± 6686 | 13,552 ± 10,927 |
| AUC_0–24h_, ng⋅h/mL | 115,983 ± 76,401 | 119,731 ± 112,601 |

*AUC_0–24h_* area under the plasma concentration–time curve over 24 hours, *C_max_* maximum plasma concentration, *C_min_* minimum plasma concentration, *GT* genotype, SD, standard deviation
^a^*n* = 8 for C_max_ and AUC_0–24h_
^b^*n* = 21 for AUC_0–24h_
