# Supplementary material for: Efficacy, safety and pharmacokinetics of simeprevir and TMC647055/ritonavir with or without ribavirin and JNJ-56914845 in HCV genotype 1 infection
Source: BMC Gastroenterol. 2017 Feb 10;17:26. doi: 10.1186/s12876-017-0580-2 (PMC5303260; doi:10.1186/s12876-017-0580-2)
Supplement: Additional file 6: — Table S4. Week-4 ritonavir pharmacokinetic parameters after administration in (a) Panels 1–3 and (b) Panel 4. (DOCX 15 kb) [file 12876_2017_580_MOESM6_ESM.docx]

**Additional file 6: Table S4** Week-4 ritonavir pharmacokinetic parameters after administration in (**a**) Panels 1–3 and (**b**) Panel 4

**a**

|  | Simeprevir 75 mg + TMC647055/ritonavir 450/30 mg | | | Simeprevir 75 mg +  TMC647055/ritonavir 600/50 mg | |
| --- | --- | --- | --- | --- | --- |
|  | Panel 1 | Panel 2 | | Panel 3 | |
| Mean ± SD | GT1a/with ribavirin (*n* = 10) | GT1b/with ribavirin (*n* = 12) | GT1b/without ribavirin (*n* = 9)^a^ | GT1a/with ribavirin (*n* = 7) | GT1b/without ribavirin (*n* = 8) |
| C_min_, ng/mL | 7.15 ± 13.7 | 2.87 ± 1.96 | BQL | 19.9 ± 20.3 | 13.5 ± 13.9 |
| C_max_, ng/mL | 148 ± 95.4 | 170 ± 73.2 | 134 ± 66.8 | 389 ± 268 | 517 ± 184 |
| AUC_0–24h_, ng⋅h/mL | 1002 ± 919 | 957 ± 392 | 776 ± 555 | 3054 ± 1951 | 2958 ± 1024 |

**b**

|  | Simeprevir 75 mg + TMC647055/ritonavir 450/30 mg + JNJ-56914845 30 mg | Simeprevir 75 mg + TMC647055/ritonavir  450/30 mg + JNJ-56914845 60 mg |
| --- | --- | --- |
|  | Panel 4 | |
| Mean ± SD | GT1a/b/other (*n* = 22)^b^ | GT1a/b/other (*n* = 22) |
| C_min_, ng/mL | 4.39 ± 3.53 | 5.07 ± 6.26 |
| C_max_, ng/mL | 141 ± 75.4 | 146 ± 112 |
| AUC_0–24h_, ng⋅h/mL | 852 ± 498 | 902 ± 754 |

*AUC_0–24h_* area under the plasma concentration–time curve over 24 hours, *BQL* below quantification limit (<2.00 ng/mL), *C_max_* maximum plasma concentration, *C_min_* minimum plasma concentration, *GT* genotype, SD, standard deviation
^a^*n* = 8 for C_max_ and AUC_0–24h_
^b^*n* = 21 for AUC_0–24h_
